# Intentional Lung Transplantation Due to ABO Incompatibility: A Case Report

**DOI:** 10.7759/cureus.51116

**Published:** 2023-12-26

**Authors:** Fabio Varón-Vega, Adriana Rincón, Leidy Prada, Eduardo Tuta-Quintero, Luis J Téllez

**Affiliations:** 1 Critical Care and Lung Transplantation Service, Fundación Neumológica Colombiana, Fundación Cardio Infantil, Bogotá, COL; 2 Pulmonology Department, Fundación Neumológica Colombiana, Bogotá, COL; 3 Epidemiology and Public Health, Universidad de La Sabana, Chía, COL; 4 Thoracic Surgery, Fundación Cardio Infantil, Bogotá, COL

**Keywords:** transplant recipient, solid organ transplant, short-term transplant outcomes, abo blood-group system, lung and cardiac transplantation

## Abstract

We present a case of a 16-year-old adolescent female with blood group O+ who was diagnosed with cystic fibrosis (CF). The patient had to be hospitalized due to septic shock and respiratory failure, and extracorporeal membrane oxygenation and mechanical ventilation were applied. Faced with high urgency, she was promptly enlisted for a lung transplant, ultimately receiving a blood group A1 deceased donor lung through rescue allocation. Bilateral incompatible lung transplantation, with parental consent, was successfully performed. The postoperative course was favorable, marked by the administration of rabbit anti-thymocyte globulin, plasmapheresis, and immunosuppression (mycophenolate, steroids, and tacrolimus) as per the prescribed protocol. Notably, the patient experienced a smooth recovery without infectious complications or humoral rejection. This case highlights the viability of lung transplantation in cases of ABO incompatibility, particularly for patients in urgent need on the transplant waiting list.

## Introduction

Lung transplant incidence has witnessed a significant upswing in recent years, with a global count surpassing 4,500 procedures in 2019 [1]. Nevertheless, this can vary among different countries, and even within the regions of each country [1,2]. In 2022 in Colombia, 1,190 transplants were carried out, showing an increase of 21.1% in the corresponding statistics [2]. Despite the efforts to increase donor availability, the number of recipients has not yet been matched [2,3]. Significantly, individuals with blood group O face a dual challenge marked by reduced transplant rates and an increased risk of mortality while awaiting transplantation [4]. This is attributed to ABO incompatibility, which triggers hyperacute rejection - a condition that can be prevented by eliminating anti-ABO hemagglutinins in the recipient before the transplant procedure [3,4]. Mitigating this alarming incongruity requires the implementation of multifaceted strategies aimed at ameliorating the shortage of available lung donors.

In this context, innovative approaches such as donation after circulatory death, and harnessing the potential of blood group-incompatible donors represent promising avenues [5-8]. These strategic interventions not only hold the potential to expand the donor pool but also address specific challenges, such as improving organ viability and increasing compatibility, thereby ushering in a new era of progress in the field of lung transplantation [5,7].

ABO-incompatible transplantation has demonstrated success in various solid organs, particularly in liver and kidney transplantation, particularly from ABO-incompatible living donors [9,10]. In the field of lung transplantation, intentional ABO incompatibility has traditionally been avoided due to concerns regarding the potential risk of antibody-mediated rejection. Limited insights into this approach have been garnered, primarily through sporadic case reports, often involving administrative errors [11-13]. There is scarce data on ABO-incompatible lung transplantation in the scientific literature [8,14]. In light of this, we aimed to describe a case of pulmonary transplantation with intentional ABO incompatibility in a critically ill patient, highlighting the positive postoperative outcomes.

The patient and her family provided informed consent for the utilization of data from the medical history.

## Case presentation

The patient was a 16-year-old female diagnosed with cystic fibrosis (CF) who had experienced five exacerbations in the past year, accompanied by an end-expiratory volume (FEV1) of 17%. Due to a greater than 80% probability of mortality, she was urgently placed on the lung transplant waiting list. Her latest hospitalization had been necessitated by pulmonary sepsis caused by pan-resistant Pseudomonas aeruginosa, leading to septic shock and acute respiratory failure. Due to refractory hypoxemia, the patient underwent invasive mechanical ventilation (IMV) and venovenous extracorporeal membrane oxygenation (ECMO-VV). The treatment involved broad-spectrum antibiotics with ceftazidime avibactam and a tracheostomy. Although her sepsis resolved, and ECMO-VV was discontinued, the patient remained reliant on mechanical ventilation. On the 90th day of hospitalization, she developed refractory hypercapnic respiratory failure.

**Figure 1:**
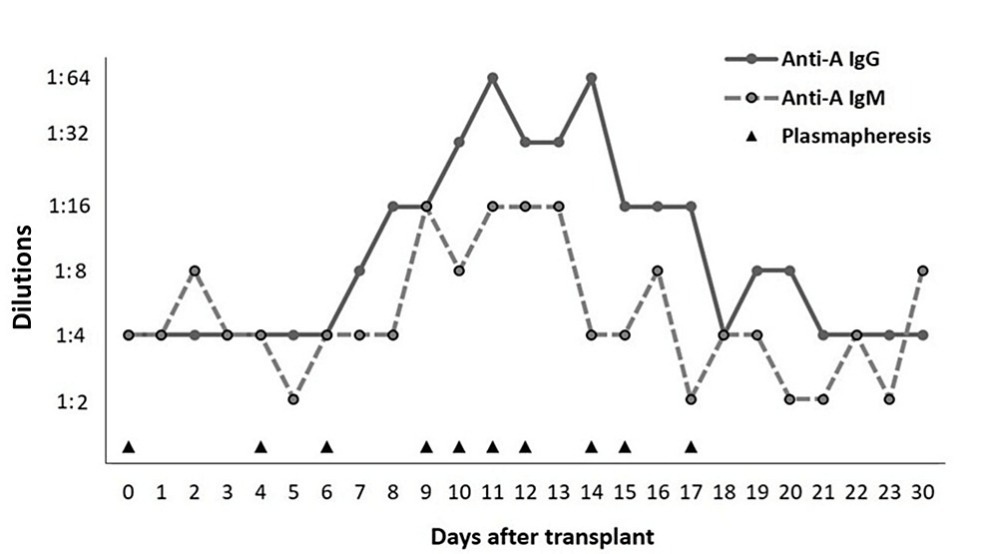
Serum titers of anti-A IgG and IgM and days of plasmapheresis

Within 10 days of inclusion on the list, an ABO-incompatible donor became available. The recipient had blood group O+, while the donor was A1+. After obtaining parental consent, an ABO-incompatible bilateral lung transplant was performed. Isoagglutinins in the recipient were negative (IgM and IgG anti-A in 1:4 dilutions). The treatment protocol included rabbit anti-thymocyte globulin at reperfusion, on the second and fourth days, amounting to a cumulative dose of 4.5mg/kg. Plasmapheresis occurred immediately post-transplant and on the fourth and sixth days. Subsequently, there was an increase in isoagglutinin titers on the eighth day, necessitating additional plasma exchange sessions until 10 sessions were completed. Figure 1 illustrates the evolution of isoagglutinin levels.

Methylprednisolone, mycophenolate, and tacrolimus were administered as per the standard institutional immunosuppression protocol. ECMO support was discontinued 24 hours post-transplant. On the fifth postoperative day, the patient experienced arterial thrombosis in the lower limbs, which was effectively addressed through embolectomy and anticoagulation with low-molecular-weight heparin. Additionally, she developed arterial hypertension associated with immunosuppressants, with no evidence of infectious complications or acute rejection at the one-month follow-up, as indicated by adequate pulmonary function tests.

## Discussion

This report aims to contribute to the body of knowledge on lung transplantation involving intentional ABO incompatibility. We describe the case of a critically ill patient who underwent a lung transplant with a satisfactory postoperative course. Our case of intentionally performed bilateral lung transplantation with ABO incompatibility bears similarity to what has been documented in the literature [8,11-14]. The prime objective in these cases is to reduce antibody titers to a level at which it is not possible to generate rejection [15]. This case adds valuable data to the existing literature and underscores the potential feasibility of intentionally ABO-incompatible lung transplantation. It is necessary to evaluate this treatment strategy implemented in our patient through clinical trials and prospective cohort studies involving patients with other incompatible solid organ transplants [9,10]. Table 1 shows the most representative differences between reported cases of lung transplant attributable to ABO incompatibility.

**Table 1:** Reported cases of lung transplantation involving ABO incompatibility BO: bronchiolitis obliterans; CF: cystic fibrosis; IPF: idiopathic pulmonary fibrosis; Tx: transplant

Study	Sex/age; diagnosis	Reason; blood group of receptor/donor	Pre-Tx receptor isoagglutinins	Treatment	Humoral rejection	Follow-up
Pierson et al., 2002 [12]	Male/67 years; IPF	Administrative error; B/A	Low titers	Anti-thymocyte globulin; antigen-specific immunoadsorption; anti-CD20 monoclonal antibody; and recombinant soluble complement receptor type 1	No	3 years (alive)
Banner et al., 2004 [11]	Female/24 years; CF	Administrative error; O/A1	1:256	Plasmapheresis. Rabbit anti-thymocyte globulin and polyspecific intravenous immunoglobulin; immunoadsorption and anti-CD20 antibody rituximab	No	128 days (alive)
Strüber et al., 2008 [14]	Female/21 years; CF	Intentional: waiting list (high urgency); O/AB	Low titers	Plasmapheresis. High‐dose intravenous immunoglobulin; anti-CD20 antibody rituximab; anti‐A and anti‐B immunoadsorption	No	9 months (alive)
Grasemann et al., 2011 [8]	Male/infant; surfactant protein B deficiency	Intentional: high probability of death; A1/B	Low titers	Intraoperative plasma exchange	No	6 months (alive)
Snell et al., 2013 [13]	Male/13 years; Re-Tx BO	Administrative error; O/B	Low titers	Plasmapheresis; anti-thymocyte globulin and intravenous immunoglobulin	No	9 years (alive)

In our case, a modification to the institutional immunosuppression protocol was implemented, incorporating plasmapheresis and rabbit anti-thymocyte globulin. The objective was to achieve isoagglutinin levels below 1:16 dilutions. Additionally, thresholds below 1:32 are deemed safe, aligning with ABO-incompatible renal transplantation algorithms [10]. Alternative antibody depletion strategies encompass immunoadsorption, splenectomy, intravenous polyclonal immunoglobulin, medications targeting antibody synthesis reduction (such as cyclophosphamide, mycophenolate, anti-thymocyte globulin, and rituximab), and complement activation inhibitors [10,12].

Data on ABO-incompatible lung transplantation is limited; however, case reports demonstrate favorable survival outcomes with no evidence of humoral rejection during follow-up [8, 12-14]. Despite the elevation in isoagglutinin titers, the clinical trajectory does not deviate from the expected course in an ABO allograft-compatible patient, thanks to adaptive changes occurring in the graft endothelium, a phenomenon known as accommodation [10]. Based on available medical literature, this process of immune accommodation may manifest approximately two weeks after the operation [10,14].

## Conclusions

Given the scarcity of donor lung organs, ABO-incompatible lung transplantation emerges as a potential option for high-urgency patients on the waiting list. We described the case of a critically ill patient who underwent such a transplant with a satisfactory postoperative course. Our case of intentionally performed bilateral lung transplantation with ABO incompatibility bears similarity to previous studies in the literature. Previous case reports have underscored the chances of success through a comprehensive approach involving conventional immunosuppression combined with antibody removal strategies. However, further research is required to ascertain the long-term prognosis of such cases and their integration into transplant center protocols.
